# Deep learning-based breast MRI for predicting axillary lymph node metastasis: a systematic review and meta-analysis

**DOI:** 10.1186/s40644-025-00863-3

**Published:** 2025-03-31

**Authors:** Chia-Fen Lee, Joseph Lin, Yu-Len Huang, Shou-Tung Chen, Chen-Te Chou, Dar-Ren Chen, Wen-Pei Wu

**Affiliations:** 1 Department of Radiology, Changhua Christian Hospital, Changhua, Taiwan; 2 Department of Biomedical Imaging and Radiological Sciences, National Yang Ming Chiao Tung University, Taipei, Taiwan; 3 Division of General Surgery, Changhua Christian Hospital, Changhua, Taiwan; 4 Comprehensive Breast Cancer Center, Changhua Christian Hospital, Changhua, Taiwan; 5 Division of Breast Surgery, Yuanlin Christian Hospital, Yuanlin, Taiwan; 6 Department of Computer Science, Tunghai University, Taichung, Taiwan; 7 Department of Medical Imaging, Changhua Christian Hospital, 135 Nanxiao Street, Changhua, 500 Taiwan

## Abstract

**Background:**

To perform a systematic review and meta-analysis that assesses the diagnostic performance of deep learning algorithms applied to breast MRI for predicting axillary lymph nodes metastases in patients of breast cancer.

**Methods:**

A systematic literature search in PubMed, MEDLINE, and Embase databases for articles published from January 2004 to February 2025. Inclusion criteria were: patients with breast cancer; deep learning using MRI images was applied to predict axillary lymph nodes metastases; sufficient data were present; original research articles. Quality Assessment of Diagnostic Accuracy Studies-AI and Checklist for Artificial Intelligence in Medical Imaging was used to assess the quality. Statistical analysis included pooling of diagnostic accuracy and investigating between-study heterogeneity. A summary receiver operating characteristic curve (SROC) was performed. R statistical software (version 4.4.0) was used for statistical analyses.

**Results:**

A total of 10 studies were included. The pooled sensitivity and specificity were 0.76 (95% CI, 0.67–0.83) and 0.81 (95% CI, 0.74–0.87), respectively, with both measures having moderate between-study heterogeneity (I^2^ = 61% and 60%, respectively; *p* < 0.01). The SROC analysis yielded a weighted AUC of 0.788.

**Conclusion:**

This meta-analysis demonstrates that deep learning algorithms applied to breast MRI offer promising diagnostic performance for predicting axillary lymph node metastases in breast cancer patients. Incorporating deep learning into clinical practice may enhance decision-making by providing a non-invasive method to more accurately predict lymph node involvement, potentially reducing unnecessary surgeries.

**Supplementary Information:**

The online version contains supplementary material available at 10.1186/s40644-025-00863-3.

## Introduction

Breast cancer is a significant global health concern, being the most commonly diagnosed cancer and the leading cause of cancer-related death among women worldwide. In 2020, there were an estimated 2.3 million new cases of female breast cancer, representing 11.7% of all cancer cases, with 685,000 deaths recorded [1]. Staging of the axillary lymph nodes (ALNs) is crucial in managing breast cancer, as it guides the clinical stages, treatment planning, and prognosis. The most precise method to assess the status of ALN in breast cancer patients is by performing a surgical lymph node biopsy, which can be performed by axillary lymph node dissection (ALND) or sentinel lymph node biopsy (SLNB) [2, 3].

The use of surgical axillary lymph node staging among older women with early breast cancer remains controversial. Based on the findings of the ongoing randomized controlled trials such as SOUND, INSEMA, BOOG 2013–08, and NAUTILUS, the omission of SLNB was safe in selected patients with small breast cancers, favorable hormone receptor-positive status, and clinically negative axillary lymph nodes [4–11]. These findings indicate that axillary surgery can be safely avoided in such patients when the absence of pathological information does not affect the postoperative treatment plan. However, despite this evidence, concerns remain among surgeons regarding these recommendations. Given the lack of survival benefit from axillary clearance in SLN-positive patients, the key question is: Should SLNB be omitted in selected low-risk patients with early breast cancer, or does it still provide enough therapeutic value to justify its routine use?

Pre-operative axillary ultrasound has a variable sensitivity, with a standard identification rate of metastatic axillary lymph nodes at 40–50% in breast cancer patients prior to SLNB [11, 12]. However, it is operator-dependent, and scanning deep areas is difficult, especially in obese patients [13, 14]. Given the MRI’s ability to evaluate all axillary LNs regardless of the depth of nodes and body status of patients, preoperative breast MRI has emerged as a valuable tool for assessing axillary lymph node metastases in breast cancer patients. At the lymph node level, MRI demonstrated the highest sensitivity at 0.70 (95% CI: 0.58–0.80) and the highest specificity at 0.89 (95% CI: 0.85–0.92) compared to ultrasound or PET-CT [15–18].

Over the past years, significant advancements in AI algorithms have transformed the medical imaging landscape, particularly in enhancing diagnostic precision and efficiency. Deep learning, distinct from traditional machine learning and radiomics, operates as an end-to-end model that automatically learns and extracts intrinsic features from medical images, eliminating human intervention and bypassing traditional, complex processes [19]. Deep learning models, particularly convolutional neural networks (CNNs), making them ideal for complex tasks like predicting axillary lymph node metastases from breast MRI [20–31]. Given the rapid advancement of deep learning applications, different AI algorithms and the target of prediction, whether the focus is on the tumor itself or the axillary lymph nodes, leading to varying accuracy.

While previous sparse systematic reviews/meta-analysis has covered AI techniques, including both machine learning and deep learning [32, 33], their findings remain sparse and lack a focused evaluation of deep learning’s potential. Therefore, this study aimed to conduct a systematic review and meta-analysis to evaluate the diagnostic accuracy of deep learning-based MRI in predicting lymph node metastases in breast cancer, offering a comprehensive assessment of its clinical efficacy.

## Materials and methods

### Study design and eligibility criteria for study selection

This study was conducted in adherence to the Preferred reporting items for systematic review and meta-analysis of diagnostic test accuracy studies (PRISMA-DTA) 2020 guidelines [34]. The PRISMA checklists are provided in supplementary material (Tables S1 and S2). The study protocol was registered with the International Platform of Registered Systematic Review and Meta-analysis Protocols (INPLASY) under the registration number INPLASY202520053.

### Search strategy and literature screening

Two authors, C.F.L. and W.P.W., with 6 and 10 years of experience in breast imaging, respectively, conducted a comprehensive literature search to gather relevant research data. We searched PubMed, MEDLINE and Embase databases for English-language articles published from January 2004 to February 2025. The predefined search terms included "artificial intelligence," "breast," "breast cancer," "lymph nodes," and "magnetic resonance imaging." The two authors independently screened titles and abstracts for eligibility and reviewed the full text of potentially relevant articles. The detailed search strategy is presented in Supplementary Text S1.

Articles were included based on the satisfaction of all the following criteria: (I) inclusion of patients diagnosed with breast cancers; (II) deep learning using breast MRI images were applied to predict axillary lymph nodes metastases; (III) sufficient data were present in terms of predictive performance of the deep learning algorithms; (IV) original research articles. Studies without enough information to calculate true positive (TP), true negative (TN), false positive (FP), and false negative (FN) values were excluded.

### Study selection, data collection, and quality assessment

Data from relevant studies were extracted and recorded in a predefined Microsoft Excel spreadsheet. For each analyzed study, the following information was collected: first author, publication year, number of patients in each dataset, specific AI algorithm model, MRI sequence used, reference standard, diagnostic accuracy, validation method, and AUC value. Diagnostic accuracy metrics, including TP, FP, TN, and FN, were either manually derived or reconstructed. These values were directly entered into contingency tables and used to calculate sensitivity and specificity. In cases where external validation was conducted, this data was prioritized. For studies that examined multiple algorithms, data from the algorithm demonstrating the best performance were extracted.

Finally, study quality assessment was performed by the same two readers according to the Checklist for Artificial Intelligence in Medical Imaging (CLAIM) and Quality Assessment of Diagnostic Accuracy Studies (QUADAS)-AI [35, 36]. Discrepancies were resolved in consensus.

### Statistical analysis

Statistical analysis was performed by the author (C.F.L.) using package meta (v 8.0–2), package lme4 (v 1.1–36), package mada (v 0.5.11), package emmeans (v 1.10.1), package rje (v 1.12.1) in R version 4.4.0 (RStudio, Boston, MA). The pooled proportion analysis of detectability estimates with 95% confidence intervals (CI) was performed using a random-effects model. This model assumes significant diversity among different studies and accounts for both intra-study sampling errors and inter-study variances. It typically provides wider confidence intervals than the fixed effects model. The predictive accuracy was quantified using pooled sensitivity, specificity with 95% CI. A *p*-value < 0.10 indicates the presence of heterogeneity. To quantify the effect of heterogeneity, the inconsistency index (I^2^) was used. I^2^ values of 25%, 50%, and 75% are interpreted as low, moderate, and high, respectively [37]. A cross-hairs plot was also generated to better display the variability in sensitivity and specificity estimates [38].

Summary receiver-operating characteristic curves using the bivariate method were constructed to display the mean value. The area under the curve (AUC) and the partial area under the curve (pAUC), which is calculated by restricting the computation of the AUC to the observed false positive rates, were also calculated. To ensure a more accurate summary estimation, weighting based on study sample size was applied, giving larger studies greater influence on the final SROC curve. An AUC of 0.9∼1 is considered excellent, 0.8∼0.9 very good, 0.7∼0.8 good, 0.6∼0.7 sufficient, and 0.5∼0.6 bad in terms of diagnostic accuracy [38].

## Results

### Literature search

**Fig. 1:**
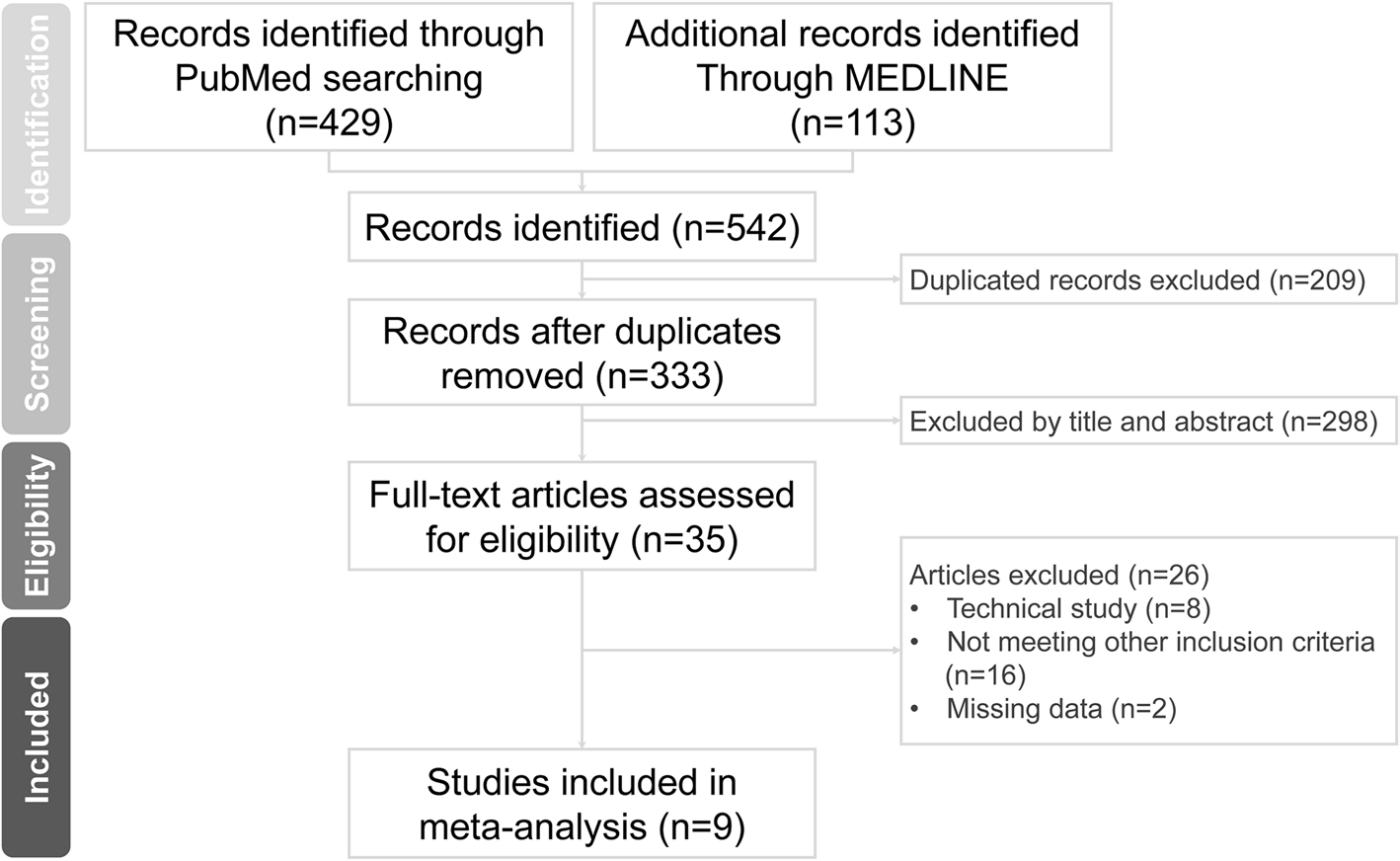
A flowchart of the literature review and study selection

Figure 1 summarizes the search and screening results for the relevant studies. Initially, we identified a total of 2667 articles through the literature review, with 524 articles found in PubMed, 205 articles from MEDLINE and 1938 articles from Embase. After removing 638 duplicate articles, 2029 unique studies remained. These records underwent screening by title and abstract, resulting in the exclusion of 1981 studies that did not meet the inclusion criteria. A total of 48 full-text articles were assessed for eligibility. During full-text reading, we excluded 38 articles because their study structures or incomplete data were not relevant to our study. Finally, 10 studies were included for meta-analysis.

### Quality assessment

The QUADAS-AI tool consists of four domains, including patient selection, index test, reference standard, and flow and timing [39]. For individual studies, each domain was assessed for applicability concerns, categorized as high, low, or unclear. Two reviewers independently conducted the quality assessments, and any discrepancies were resolved by a third reviewer to achieve consensus. Most studies were rated as the unclear risk. The detailed risk of bias information on each enrolled study can be found in supplemental Fig. 1.

The meta-analysis of the quality assessment, based on the CLAIM criteria and presented in Table S3, revealed an average total score of 34.1 across these studies, with a standard deviation of 3.67 [40]. This indicates a moderate level of variability in the overall quality of the included research.

### Basic characteristics of the included literature

The characteristics of each eligible study are detailed in Table 1. All studies used a retrospective design, six involving internal validation [24–27, 30, 31] and four with external validation [23, 28, 29, 41].

**Table 1:** Characteristics of the included studies

Studies	N of Patients	AI Algorithm	MRI Sequences	Reference Standard	Validation method	External Validation	AUC	TP	FP	FN
Nguyen, 2020 [31]	357	CNN	DCE-MRI	Histopathology	Nested, stratified group tenfold cross-validation	No	0.71	10	10	4
Ren, 2020 [25]	99	CNN	T1WI (post contrast)	^18^FDG-PET	fivefold cross-validation	No	0.91	20	6	2
Ren, 2022 [26]	56	CNN	T1WI + T2WI	^18^FDG-PET	threefold cross-validation	No	0.88	20	4	5
Santucci, 2022 [24]	153	CNN	DCE-MRI	Histopathology	tenfold cross-validation	No	0.77	4	2	1
Zhang, 2022 [30]	252	ResNet50	Multiparametric MRI model	Histopathology	Split training-test	No	0.91	17	3	3
Gao, 2023 [23]	941	ResNet, RCNet, SVM	DCE-MRI	Histopathology	fivefold cross-validation	Yes	0.85	38	10	10
Guo, 2024 [28]	2063	ConvRNN, CNN	DCE-MRI	Histopathology	Split training-test	Yes	0.81	45	11	35
Polat, 2024 [27]	350	CNN	DCE-MRI	MRI, axillary US, US-guided needle biopsy, Histopathology	Nested fivefold cross-validation	No	0.87	27	5	3
Zhou, 2024 [29]	1259	DLWPS (ResNet101, ResNeXt101, DenseNet)	DCE-MRI	Histopathology	fivefold cross validation	Yes	0.92	29	11	9

*AUC *area under the receiver operating curves, *SEN *sensitivity, *SPE *specificity, *PPV *positive predictive value, *NPV *negative predictive value, *CNN *convolutional neural network, *RCNet *ResNet and convolutional block attention module, *2D ConvRNN *convolutional neural network and recurrent neural network, *DLWPS *deep learning-based whole process system, *ResNeXt101 *modified version of ResNet101, *DCE-MRI *dynamic contrast-enhanced magnetic resonance imaging, *T1WI *T1-weighted imaging, *T2WI *T2-weighted imaging, *DWI *diffusion-weighted imaging

Most studies used the primary tumor as the target lesion for predicting axillary lymph node metastases. However, three studies included both the primary tumor and axillary lymph nodes as targets [23, 25, 26].

Seven of these studies reported the best model performance using DCE-MRI images [23–25, 27–29, 31, 41]. In 10 eligible studies, different AI algorithms were used for modeling. Two of these studies incorporated clinical information in addition to images [27, 31], and one used ensemble learning [30]. Most studies identified lymph node metastases through pathological examination, including surgical resection or needle biopsy. However, one study relied on clinical node status [31] and two studies employed ^18^FDG-PET for indirect assessment [25, 26].

### Diagnostic accuracy and heterogeneity

**Fig. 2:**
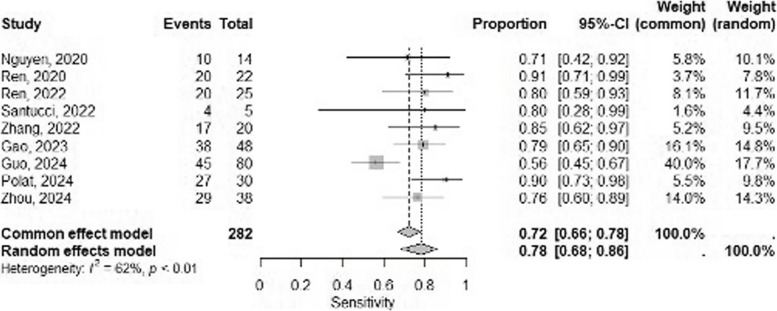
Forest plots of pooled sensitivity (a) and specificity(b) for the deep learning model to detect axillary lymph node metastasis in breast cancer

**Fig. 3:**
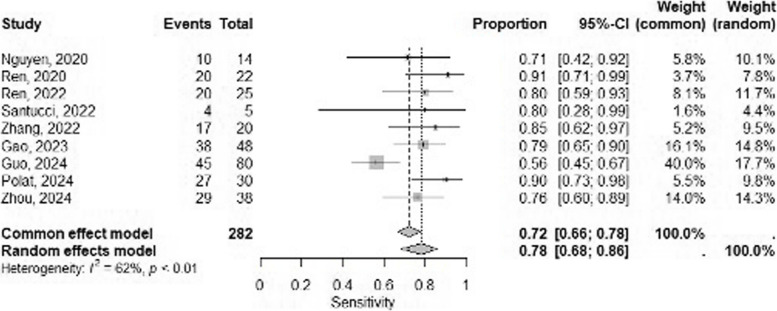
Forest plots of the sensitivity (a) and specificity (b) for subgroup analysis according to the target of assessment (the primary tumor only or the axillary lymph nodes and the primary tumor)

Figure 2 presents forest plots illustrating sensitivities (Fig. 2a) and specificities (Fig. 2b), each with the corresponding 95% confidence intervals. The overall pooled sensitivity and specificity were 0.76 (95% CI, 0.67–0.83) and 0.81 (95% CI, 0.74–0.87), respectively, with both measures having moderate between-study heterogeneity (I^2^ = 61% and 60%; *p* < 0.01 and* p* < 0.01, respectively). In the subgroup analysis, studies using only the primary tumor as the target reported an overall pooled sensitivity of 0.73 (95% CI, 0.61–0.82) and specificity of 0.78 (95% CI, 0.69–0.86). In contrast, studies using both the axillary lymph nodes and the primary tumor as targets showed slightly higher overall pooled sensitivity and specificity of 0.81 (95% CI, 0.72–0.88) and 0.86 (95% CI, 0.77–0.92), respectively, as illustrated in Fig. 3.

**Fig. 4:**
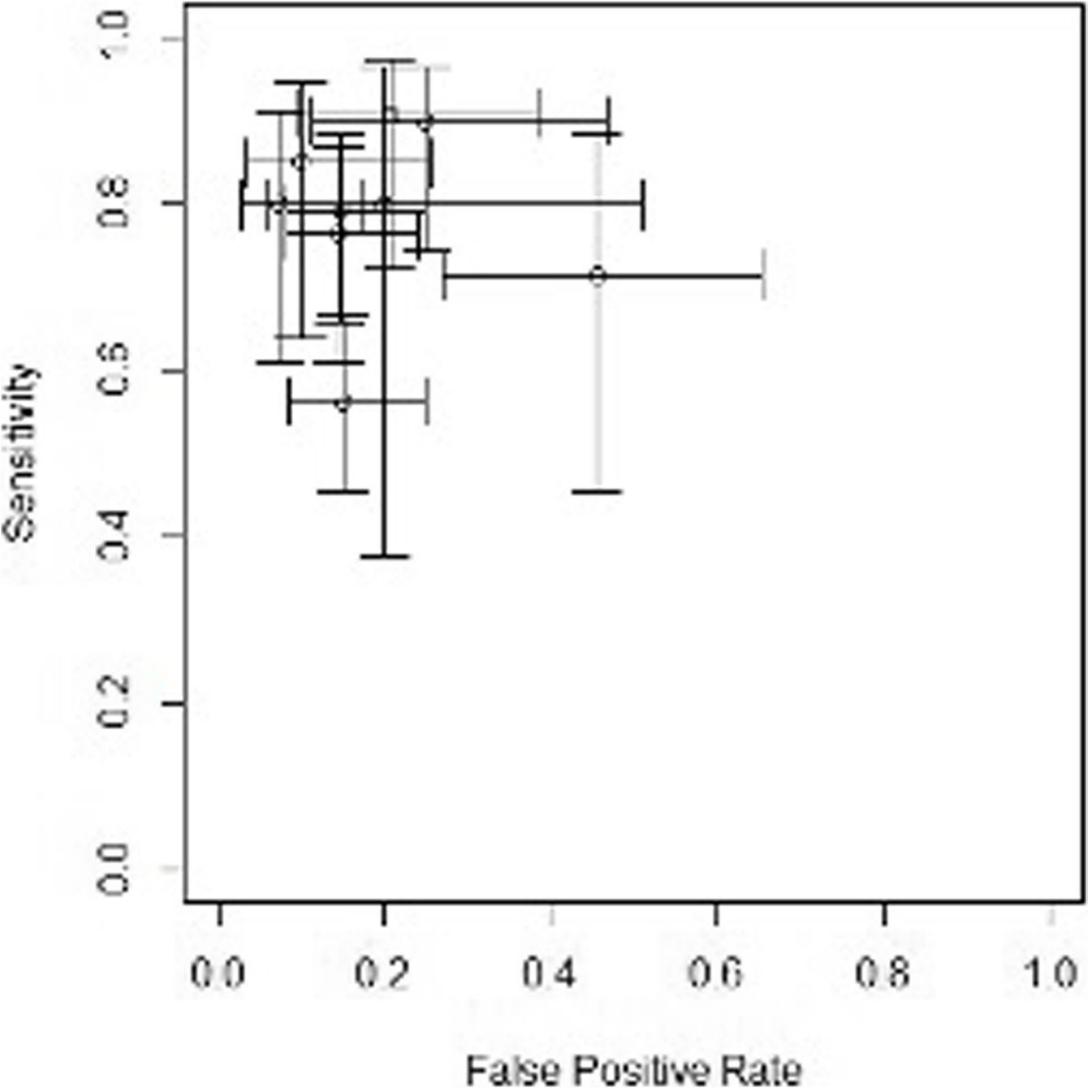
Cross-hair plot of studies included in the meta-analysis

Figure 4 depicts a cross-hair plot that visualizes the sensitivity and specificity estimates for each study included in the meta-analysis.

**Fig. 5:**
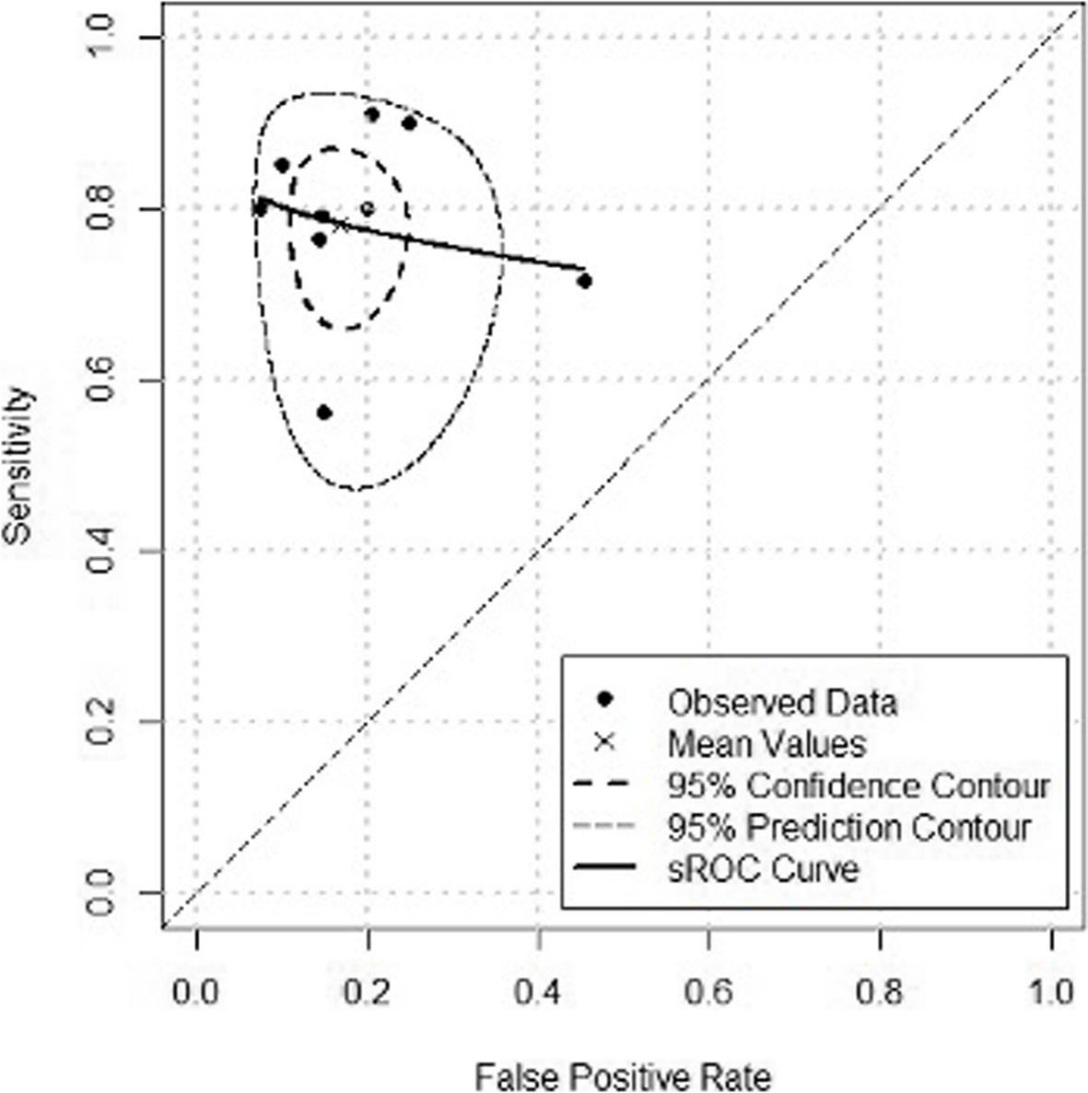
Summary receiver operating characteristic (SROC) curves based on the bivariate approach

The pooled SROC curve by the bivariate approach showed good diagnostic accuracy with a weighted AUC of 0.79 and a weighted pAUC value of 0.76. The Diagnostic Odds Ratio (DOR) across studies was 14.7 (95% CI 7.6–28.3), with heterogeneity observed at 64.1% (I^2^ = 64.%, *p* = 0.003), suggesting moderate to high heterogeneity (Fig. 5).

## Discussion

The transition from traditional machine learning to deep learning marks a major milestone in the evolution of artificial intelligence, enabling more complex data-driven decision-making and predictive analytics across various domains, particularly in medical imaging [42]. Deep learning leverages deep neural networks with multiple layers to automatically analyze complex and diverse data [43]. This advancement has driven significant progress in areas such as image processing and predictive modeling, highlighting deep learning’s pivotal role in personalized treatments and diagnostic tools to identify diseases more accurately and quickly.

This meta-analysis is to evaluate the diagnostic performance of deep learning-based algorithms applied to breast MRI for predicting lymph node metastasis in breast cancer patients. Breast MRI is increasingly recognized as a crucial imaging modality for pre-operative staging [44]. However, variations in image quality and the subjectivity of radiological interpretation can lead to false positives and false negatives, potentially resulting in unnecessary surgeries for breast cancer patients. From the radiologists' perspective, the role of breast MRI in determining axillary lymph nodes metastases has shown moderate sensitivity and negative predictive value, but only low-to medium specificity [45]. This can lead to either overestimation or underestimation of nodal staging (N staging). Therefore, a non-invasive and highly accurate method to predict axillary lymph node metastasis before surgery is essential to guide clinical decision-making effectively.

Our meta-analysis demonstrated promising results for axillary lymph nodes metastases classification, with a pooled sensitivity and specificity of 76% and 81%, respectively. The diagnostic performance by radiologists reported sensitivities of 77% and 55% and specificities of 90% and 86%, as demonstrated in the meta-analysis by Zhou et al. [17](2018) and Zhang et al. [46](2020). Meanwhile, machine learning-based models have shown significant potential with pooled sensitivities and specificities ranging from 76 to 85% and 81% to 83% in the meta-analysis by Zhang et al. [47](2022), Chen et al. [33] (2021), and Liu et al. [32] (2023). These findings indicate that deep learning-based models offer comparable, if not superior, diagnostic performance compared to traditional radiologist interpretations and other machine learning models to detect axillary lymph node metastasis (ALNM) in breast cancer patients.

To date, many meta-analyses have been performed to evaluate the diagnostic performance of breast MRI for ALNM. Chen et al. found that MRI sequences and algorithms as key factors influencing the diagnostic accuracy of machine learning-assisted MRI [33]. Our subgroup analysis revealed that studies targeting both the primary tumor and axillary lymph nodes demonstrated better diagnostic performance compared to those focusing solely on the primary tumor. Factors such as differences in study populations, imaging protocols, image segmentation methods (eg, included breast lesion or lymph nodes) and model architectures may contribute to this heterogeneity and should be carefully addressed in future research.

A deep learning-based algorithm can enhance the speed of image interpretation for detecting lymph node metastases in breast cancer and is likely to be integrated alongside clinical judgment to optimize decision-making. However, challenges and limitations remain. With a pooled sensitivity and specificity of approximately 0.80, the algorithm could potentially miss 1 in 5 women with lymph node metastases, which may affect critical treatment decisions, such as the need for surgery or neoadjuvant chemotherapy. On the other hand, the model correctly identifies most patients without axillary lymph node metastases, helping to minimize unnecessary interventions, such as surgical lymph node dissection or overtreatment.

Moving forward, while the findings of this meta-analysis are promising, several considerations should be taken into account. First, the heterogeneity observed in both sensitivity and specificity underscores the need for further investigation into the sources of variability across studies. The integration of deep learning in medical imaging brings several ethical challenges that must be addressed to ensure safe and equitable use. Deep learning models trained on limited or region-specific datasets has the possibility to produce biased results, leading to reduced diagnostic accuracy in underrepresented populations. There were relatively few deep learning studies eligible for inclusion. Due to incomplete reporting of results in few studies, estimates of diagnostic performance were derived from a limited data set. Finally, the diversity in scanner types, imaging protocols, and criteria for defining lymph node metastasis across studies may have influenced the accuracy of the results.

## Conclusions

In conclusion, our meta-analysis demonstrates that deep learning-based MRI offers promising diagnostic performance for predicting axillary lymph node metastases in breast cancer. These results suggest that deep learning models have the potential to complement radiologists, enhance diagnostic accuracy, and reduce unnecessary surgical interventions. However, variability among studies highlights the need for standardized imaging protocols and larger, multicenter studies to further validate their clinical utility. Future research should focus on integrating deep learning with clinical and genomic data to enable more personalized and precise treatment planning, ultimately improving patient outcomes.

## Supplementary Information

Additional file 1

Additional file 2

Additional file 3

## Data Availability

No datasets were generated or analysed during the current study.
