# Supplementary material for: Deep learning-based breast MRI for predicting axillary lymph node metastasis: a systematic review and meta-analysis
Source: Cancer Imaging. 2025 Mar 31;25:44. doi: 10.1186/s40644-025-00863-3 (PMC11956454; doi:10.1186/s40644-025-00863-3)

Figure S1. Quality assessment of included articles for the risk of bias and concerns for applicability using the QUADAS-AI criteria.


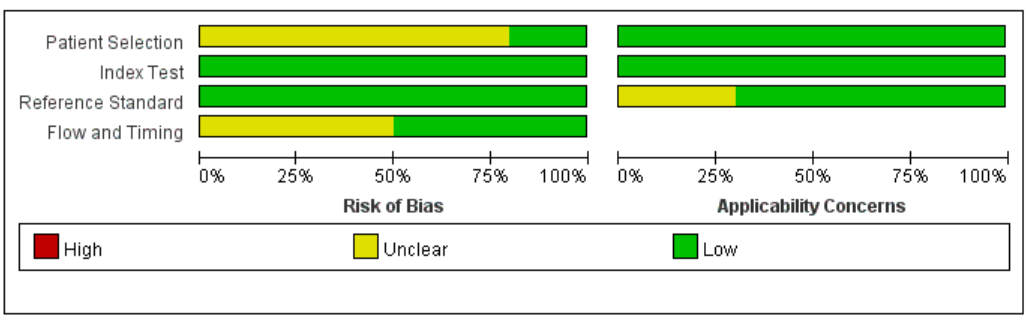


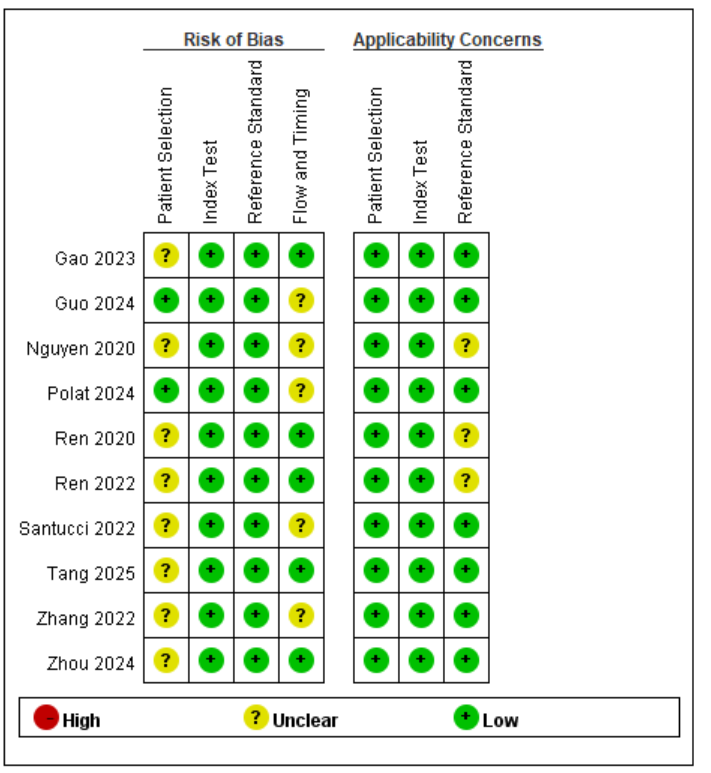

Supplement: Supplementary file 1 — Additional file 1 [file 40644_2025_863_MOESM1_ESM.docx]
