# Supplementary material for: Deep learning-based breast MRI for predicting axillary lymph node metastasis: a systematic review and meta-analysis
Source: Cancer Imaging. 2025 Mar 31;25:44. doi: 10.1186/s40644-025-00863-3 (PMC11956454; doi:10.1186/s40644-025-00863-3)
Supplement: Supplementary file 2 — Additional file 2 [file 40644_2025_863_MOESM2_ESM.docx]

**Supplementary Text 1: Search Strategy**

| Sources | Search  in | Search terms | Limits | Search  results |
| --- | --- | --- | --- | --- |
| Pubmed | N/A | (("Deep Learning"[Mesh] OR "Machine Learning"[Mesh] OR "Artificial Intelligence"[Mesh] OR "Neural Networks, Computer"[Mesh] OR "deep learning" OR "machine learning" OR "artificial intelligence" OR "convolutional neural network" OR "CNN")) AND (("Breast Neoplasms"[Mesh] OR "Magnetic Resonance Imaging"[Mesh] OR "breast cancer" OR "breast carcinoma" OR "breast tumor" OR "breast neoplasm" OR "MRI")) AND (("Lymphatic Metastasis"[Mesh] OR "Lymph Nodes"[Mesh] OR "Sentinel Lymph Node"[Mesh] OR "lymphatic metastasis" OR "lymph nodes" OR "sentinel lymph node" OR "axillary lymph node" OR "axillary lymph node metastasis" OR "lymph node metastasis")) | (2004-2025),  English | 524 |
| MEDLINE | Advanced Search | (exp Deep Learning/ OR exp Machine Learning/ OR exp Artificial Intelligence/ OR exp Neural Networks, Computer/)  AND (exp Breast Neoplasms/ OR exp Magnetic Resonance Imaging/)  AND (exp Lymphatic Metastasis/ OR exp Lymph Nodes/ OR exp Sentinel Lymph Node/) | (2004-Current),  English | 205 |
| Embase | Advanced Search | ('deep learning'/exp OR 'machine learning'/exp OR 'artificial intelligence'/exp OR 'neural network'/exp OR 'convolutional neural network'/exp OR 'cnn'/exp) AND  ('breast cancer'/exp OR 'magnetic resonance imaging'/exp OR 'breast carcinoma'/exp OR 'breast tumor'/exp OR 'breast neoplasm'/exp OR 'mri'/exp) AND  ('lymphatic metastasis'/exp OR 'lymph node'/exp OR 'sentinel lymph node'/exp OR 'axillary lymph node'/exp OR 'axillary lymph node metastasis'/exp OR 'lymph node metastasis'/exp) | (2004-2025),  English | 1938 |
