# Supplementary material for: Deep learning-based breast MRI for predicting axillary lymph node metastasis: a systematic review and meta-analysis
Source: Cancer Imaging. 2025 Mar 31;25:44. doi: 10.1186/s40644-025-00863-3 (PMC11956454; doi:10.1186/s40644-025-00863-3)
Supplement: Supplementary file 3 — Additional file 3 [file 40644_2025_863_MOESM3_ESM.zip › Table S1.docx]

**Table S1. The PRISMA-DTA for Abstract Checklist.**

| Section/Topic | Item no. | PRISMA-DTPA for Abstracts Checklist Item | Reported on Page# |
| --- | --- | --- | --- |
| **Title and purpose** | | |  |
| Title | 1 | Identify the report as a systematic review (+/− meta-analysis) of diagnostic test accuracy studies. | 1 |
| Objectives | 2 | Indicate the research question, including components such as participants, index test, and target conditions. | 3 |
| **Methods** | | |  |
| Eligibility criteria | 3 | Include study characteristics used as criteria for eligibility. | 3 |
| Information sources | 4 | List the key databases searched and the search dates. | 3 |
| Risk of bias and applicability | 5 | Indicate the methods of assessing risk of bias and applicability. | 3 |
| Synthesis of results | A1 | Indicate the methods for the data synthesis. | 3 |
| **Results** | | |  |
| Included studies | 6 | Indicate the number and type of included studies and the participants and relevant characteristics of the studies (including the reference standard). | 3 |
| Synthesis of results | 7 | Include the results for the analysis of diagnostic accuracy, preferably indicating the number of studies and participants. Describe test accuracy including variability; if meta-analysis was done, include summary results and confidence intervals. | 3 |
| **Discussion** | | |  |
| Strengths and limitations | 9 | Provide a brief summary of the strengths and limitations of the evidence. | 4 |
| Interpretation | 10 | Provide a general interpretation of the results and the important implications. | 4 |
| **Other** | | |  |
| Funding | 11 | Indicate the primary source of funding for the review. | NA |
| Registration | 12 | Provide the registration number and the registry name. | NA |

NA, not applicable.

Adapted From: McInnes MDF, Moher D, et al. The PRISMA-DTA Group (2018). Preferred Reporting Items for a Systematic Review and Meta-analysis of Diagnostic Test Accuracy Studies: The PRISMA-DTA Statement. JAMA. 2018 Jan 23;319(4):388-396. doi: 10.1001/jama.2017.19163.
