# Supplementary material for: Deep learning-based breast MRI for predicting axillary lymph node metastasis: a systematic review and meta-analysis
Source: Cancer Imaging. 2025 Mar 31;25:44. doi: 10.1186/s40644-025-00863-3 (PMC11956454; doi:10.1186/s40644-025-00863-3)
Supplement: Supplementary file 3 — Additional file 3 [file 40644_2025_863_MOESM3_ESM.zip › Table S3.docx]

**Table S3. The Checklist for Artificial Intelligence in Medical Imaging scores.**

| Source | Title/Abstract | Introduction | Methods | | | | | | | Results | | Discussion | Other Information | Total Score |
| --- | --- | --- | --- | --- | --- | --- | --- | --- | --- | --- | --- | --- | --- | --- |
|  |  |  | Study design | Data | Ground truth | Data preparation | Model | Training | Evaluation | Data | Model performance |  |  |  |
|  | (2) | (2) | (2) | (7) | (5) | (3) | (3) | (3) | (5) | (2) | (3) | (2) | (3) | (42) |
| Nguyen, 2020^27^ | 2 | 2 | 2 | 5 | 4 | 2 | 2 | 2 | 3 | 2 | 2 | 2 | 1 | 31 |
| Ren, 2020^21^ | 2 | 2 | 2 | 5 | 4 | 2 | 2 | 2 | 2 | 2 | 2 | 2 | 1 | 30 |
| Ren, 2022^22^ | 2 | 2 | 2 | 5 | 5 | 3 | 3 | 2 | 3 | 2 | 2 | 1 | 3 | 35 |
| Santucci, 2022^20^ | 2 | 2 | 2 | 5 | 4 | 2 | 2 | 2 | 2 | 2 | 2 | 2 | 1 | 30 |
| Zhang, 2022^26^ | 2 | 2 | 2 | 5 | 4 | 2 | 2 | 3 | 2 | 1 | 2 | 2 | 1 | 30 |
| Gao, 2023^19^ | 2 | 2 | 2 | 6 | 5 | 2 | 2 | 2 | 5 | 2 | 2 | 2 | 1 | 35 |
| Guo, 2024^24^ | 1 | 2 | 2 | 6 | 4 | 2 | 3 | 3 | 5 | 2 | 2 | 2 | 1 | 35 |
| Polat, 2024^23^ | 2 | 2 | 2 | 7 | 4 | 2 | 3 | 3 | 5 | 2 | 3 | 2 | 3 | 40 |
| Zhou, 2024^25^ | 2 | 2 | 2 | 6 | 4 | 2 | 3 | 3 | 5 | 2 | 2 | 2 | 3 | 38 |
| Tang, 2025 | 2 | 2 | 2 | 5 | 4 | 2 | 3 | 3 | 5 | 2 | 2 | 2 | 3 | 37 |

Adapted From: Mongan J, Moy L, Kahn CE, Jr. Checklist for Artificial Intelligence in Medical Imaging (CLAIM): A Guide for Authors and Reviewers. Radiol Artif Intell 2020;2(2):e200029. doi: 10.1148/ryai.2020200029 [published Online First: 20200325]
